# Aggregation and Toxicology of Titanium Dioxide Nanoparticles

**DOI:** 10.1289/ehp.10915

**Published:** 2008-04

**Authors:** Philippe Baveye, Magdeline Laba

**Affiliations:** SIMBIOS Centre, University of Abertay Dundee, Dundee, Scotland, E-mail: P.Baveye@Abertay.ac.uk; Department of Civil and Environmental Engineering, Cornell University, Ithaca, New York, E-mail: ml49@cornell.edu

In their study of inhalation exposure of titanium dioxide particles, Grassian et al. (2007) presented a transmission electron micrograph (TEM) (their Figure 2A) as an image of “dispersed” TiO_2_ nanoparticles. Yet, the TiO_2_ nanoparticles in this TEM do not appear to be dispersed. There is clear evidence of self-organization of the nanoparticles into distinct assemblages, separated by relatively large regions devoid of any particle. This spatial pattern, very unlikely to occur randomly, is even more apparent when Grassian et al.’s TEM is contrast-enhanced, sharpened, and thresholded (Figure 1A) to eliminate the initial grainy background. With this image, one can demonstrate quantitatively the extent of clustering by calculating the radial distribution function (Torquato 2002), defined as the probability of finding a nanoparticle, in any direction, at various distances away from the center of a given nanoparticle. We compared the values obtained for this function with those associated with an image in which the same nanoparticles have been artificially dispersed (with image processing software). In the dispersed case (Figure 1B), the probability of finding a black pixel drops precipitously when the distance exceeds the apparent radius of nanoparticles, and then stays close to zero thereafter. In the “original” case (Grassian et al.’s Figure 2A), there is also a drop, but the radial distribution function never gets to zero. It progressively increases again as the radial distance increases. This quantitative difference between the curves in Figure 1B leads to the conclusion that the nanoparticles in Figure 1A are clustered.

However, this conclusion is intriguing in itself. Indeed, before obtaining their TEM, Grassian et al. (2007) suspended the TiO_2_ nanoparticles in methanol and sonicated the suspension for an unspecified, but presumably appreciable “period of time.” Given this strongly dispersive treatment, it is remarkable that aggregation still occurred to the extent it did. This observation suggests that the 2- to 5-nm size of the primary TiO_2_ “nano”-particles may be somewhat irrelevant to environmental and toxicologic concerns because in nature, under conditions far more conducive to aggregation than those imposed by Grassian et al. (2007), nanoparticles may never be found alone, but are part of significantly larger-sized aggregates. In a recent study, French et al. (French RA, Jacobson AR, Kim B, Isley SL, Penn RL, Baveye PC, unpublished data) observed that in aqueous suspensions under a range of environmentally relevant conditions of pH and ionic strength, TiO_2_ nanoparticles form aggregates of several hundred nanometers to several micrometers in diameter within minutes.

This aggregation may have toxicologic implications. In any given system (e.g., aerosols), it is possible that even a slight change in pH or ionic strength may cause TiO_2_ nanoparticles to cluster differently, and therefore to have very dissimilar biological activity. In general, this might explain mixed results found in the literature on the toxicity of TiO_2_ nanoparticles to environmentally relevant species. Until now, these inconclusive results have been explained (Oberdörster et al. 2005) by arguing that the high biological activity of TiO_2_ nanoparticles, caused by their large specific surface area, creates a high potential for inflammatory, pro-oxidant, and antioxidant activity. Yet, conflicting observations may perhaps be imputable instead to compounding factors due to nanoparticle aggregation, which so far has not been given serious consideration.

## Figures and Tables

**Figure 1:**
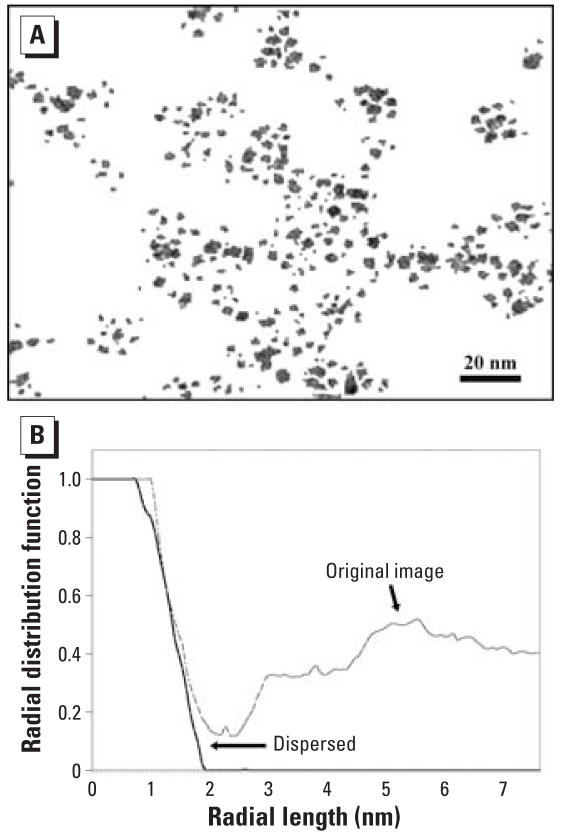
(*A*) Contrast-enhanced, sharpened, and segmented version of a TEM of a TiO_2_ nanoparticle suspension (modified from Grassian et al. 2007). (*B*) Radial distribution function versus radial distance for a representative point in a nanoparticle in (*A*); the dashed line indicates values for the “original image” [Figure 2A from Grassian et al. (2007)] and the solid line represents a similar point in an image where the nanoparticles are artificially dispersed.
